# 

**Published:** 1905-06

**Authors:** 


### BOOKS RECEIVED.

Surgical Diagnosis. A Manual for Practitioners of Medicine and Surgery. By Otto G. T. Kiliani, M.D., Surgeon to the German Hospital, New York. Octavo, pp. xviii-449. Illustrated by 59 full-page plates and engravings. New York: William Wood & Co. 1905. (Price: cloth, $4.50; p. m., $5.50 net.)

Acute Contagious Diseases. By William M. Welch, M.D., Consulting Physician to the Municipal Hospital for Contagious and Infectious Diseases; Diagnostician to the Bureau of Health, etc., Philadelphia, and Jay F. Schamberg, A.B., M.D., Professor of Dermatology and of Infectious Eruptive Diseases, Philadelphia Polyclinic; Consulting Physician to the Municipal Hospital for Contagious and Infectious Diseases, and Assistant Diagnostician to the Philadelphia Bureau of Health, etc. Octavo, 781 pages. Illustrated with 109 engravings and 61 full-page plates. Philadelphia and New York: Lea Brothers & Co. 1905. (Cloth, $5.00; leather, $6.00; half morocco, $6.50 net.)

Practical Problems of Diet and Nutrition. By Max Einhorn, M.D., Professor of Medicine at the New York Post-Graduate Medical School and Hospital and Visiting Physician to the German Hospital, New York.

Octavo, pp. 64. New York: William Wood & Co. 1905. (Price, 75 cents.)

A System of Physiologic Therapeutics. A Practical Exposition of the Methods, other than Drug-giving, useful in the Treatment of the Sick and in the Prevention of Disease. Edited by Solomon Solis Cohen, A.M., M.D., Professor of Clinical Medicine at Jefferson Medical College, Philadelphia. Vol. XI. Serum-Therapy; Organotherapy; Blood-letting, etc.; Radium; Principles of Therapeutics; *X*-ray Therapy; Digest and Therapeutic Index to all volumes. By Joseph McFarland, M.D., Philadelphia; O. T. Osborne, M.D., New Haven; Frederick A. Packard, M.D., Philadelphia; Samuel G. Tracy, M.D., New York; The Editor, and R. Max Goepp, M.D., Philadelphia. Octavo, pp. 388. Illustrated. Philadelphia: P. Blakiston's Son & Co. 1905. (Complete set, cloth, $27.50 net.)

Maternitas. Care of Prospective Mother and her Child. By Charles E. Paddock, M.D., Professor of Obstetrics, Chicago Post-Graduate Medical School. Duodecimo, pp. 189. Chicago: Cloyd J. Head & Co. 1905. (Price, $1.25.)

